# Upregulation of the aging related LMNA splice variant progerin in dilated cardiomyopathy

**DOI:** 10.1371/journal.pone.0196739

**Published:** 2018-04-27

**Authors:** Moritz Messner, Santhosh Kumar Ghadge, Valentina Goetsch, Andreas Wimmer, Jakob Dörler, Gerhard Pölzl, Marc-Michael Zaruba

**Affiliations:** Medical University Innsbruck, Department of Internal Medicine III, Cardiology and Angiology, Innsbruck, Tirol, Austria; Southern Illinois University School of Medicine, UNITED STATES

## Abstract

**Background:**

Mutations in the LMNA gene are a common cause (6–8%) of dilated cardiomyopathy (DCM) leading to heart failure, a growing health care problem worldwide. The premature aging disease Hutchinson-Gilford syndrome (HGPS) is also caused by defined mutations in the LMNA gene resulting in activation of a cryptic splice donor site leading to a defective truncated prelamin A protein called progerin. Low levels of progerin are expressed in healthy individuals associated with ageing. Here, we aimed to address the role of progerin in dilated cardiomyopathy.

**Methods and results:**

mRNA expression of progerin was analyzed in heart tissue of DCM (n = 15) and non-failing hearts (n = 10) as control and in blood samples from patients with DCM (n = 56) and healthy controls (n = 10). Sequencing confirmed the expression of progerin mRNA in the human heart. Progerin mRNA levels derived from DCM hearts were significantly upregulated compared to controls (1.27 ± 0.42 vs. 0.81 ± 0.24; p = 0.005). In contrast, progerin mRNA levels in whole blood cells were not significantly different in DCM patients compared to controls. Linear regression analyses revealed that progerin mRNA in the heart is significantly negatively correlated to ejection fraction (r = -0.567, p = 0.003) and positively correlated to left ventricular enddiastolic diameter (r = 0.551, p = 0.004) but not with age of the heart per se. Progerin mRNA levels were not influenced by inflammation in DCM hearts. Immunohistochemistry and Immunofluorescence analysis confirmed increased expression of progerin protein in cell nuclei of DCM hearts associated with increased TUNEL+ apoptotic cells.

**Conclusion:**

Our data suggest that progerin is upregulated in human DCM hearts and strongly correlates with left ventricular remodeling. Progerin might be involved in progression of heart failure and myocardial aging.

## Introduction

Age is a major cardiovascular risk factor including cardiovascular disease. Therefore, elucidating aging-related processes might lead to the identification of novel treatment options for heart failure, which has a prevalence of 1–2% in the adult population in developed countries and is a growing health care problem worldwide [1, 2]. Premature aging-like syndromes like Hutchinson-Gilford progeria syndrome (HGPS) have been investigated to achieve a better understanding of pathophysiological aging processes. HGPS is based on mutations affecting the proper encoding and further processing of lamin A an important protein in the nucleus of eukaryotic cells [3] resulting in misprocessed lamin A (progerin) which also plays an important role in normal ageing [4]. Lamin A is an intermediate filament protein which is involved in forming a filamentous meshwork between the chromatin and the nuclear membrane. It is very important to keep the nuclear envelope upright [5] regulating important processes like DNA replication, DNA repair and RNA transcription [6, 7]. Moreover, LMNA mutations are a common cause (ca. 6–8%) of dilated cardiomyopathy (DCM), a major reason of severe heart failure [8–10]. In normal cells prelamin A is proteolytically cleaved by the zinc-metalloproteinase (ZMPSTE24) at the carboxyl-terminus (CAAX box) to finally result in the production of unfarnesylated lamin A [11, 12]. In 90% of the cases in HGPS a de novo point mutation in the LMNA A gene (LMNA 1824 C>T, G608G) results in a single C to T (1824) nucleotide substitution in exon 11 activating a cryptic splicing site [13], which results in the production of a truncated prelamin A protein (LMNA Δ50 aa), also called progerin. Progerin lacks the amino acids 607 to 656 including the CAAX box–the cleavage site for ZMPSTE24 [3, 14]. Consequently, the protein remains farnesylated and cannot be processed to functional lamin A, causing structural and functional nuclear abnormalities [15]. With time proceeding progerin accumulates in the nucleus and not only alters the structure of the nuclear lamina but also negatively influences the stiffness and mechanochemical properties of the nucleus [16–18]. Patients with HGPS develop severe cardiovascular morbidities like atherosclerosis and heart failure and die as teenagers due to stroke or myocardial infarction [19, 20]. Toward the end of life HGPS patients suffer from cardiomegaly and cardiac dilatation [21]. It has been shown that low levels of progerin are expressed in non HGPS-cells and that a positive correlation exists between accumulation of progerin in the nucleus and the process of ageing [4, 22]. However, the role of progerin in human DCM has never been investigated so far. Therefore, we aimed to address the role of progerin in DCM.

## Methods

### Tissue samples and patients

Ventricular tissue samples were either obtained by endomyocardial biopsy (EMB) or as dispensable tissue during heart surgery and transplantation from patients with DCM (n = 15) and non-failing control hearts (n = 10). EMB were performed in the septal-apical region of the right or left ventricle. Samples were immediately immersed in RNAlater (Qiagen GmbH) and stored at 4°C for 24 hours (RNA later incubation time) followed by long-term storage at -80°C for further RNA processing. Additionally, blood samples were obtained from patients with DCM (n = 56), and healthy controls (n = 10). Heart and blood samples were analyzed for quantitative expression of progerin and lamin A mRNA. Patients with acute heart failure, occlusive coronary artery disease on coronary angiography, valvular heart disease and moderate to severe chronic kidney disease were excluded. The diagnosis of chronic heart failure (CHF) was based on the presence of current or previous symptoms or characteristic clinical signs and evidence of left ventricular dysfunction and dilation according to current guidelines (ESC 2016) [23]. Eligible patients were ≥18 years of age. Patients were treated according to prevailing CHF guidelines [23]. In 6 patients DCM was attributed to a history of inflammatory heart disease. Diagnosis of inflammatory cardiomyopathy was based on the presence of >14 infiltrating CD45+ leukocytes/mm^2^ and/or the presence of more than 7 CD3-positive lymphocytes per mm^2^ [24]. No specific etiology was found in the remaining nine patients. The study conformed to the principles outlined in the Declaration of Helsinki and was approved by the local ethics committee of Innsbruck Medical University. All patients gave written informed consent for participation in the study.

### Echocardiography

All measurements were performed using a sonography unit (Sequoia 256, Acuson-Siemens Medical Solutions) equipped with a 3.5–1.75–MHz transducer by experienced echocardiographers. Left ventricular (LV) interventricular septum, LV posterior wall thickness, left ventricular enddiastolic diameter (LVEDD) and left ventricular endsystolic diameter (LVESD) were measured by M-mode imaging. Left ventricular ejection fraction (EF) was calculated by modified Simpson's biplane method.

### RT-PCR of mRNA

Total mRNA was extracted from heart tissue utilizing Trizol (Invitrogen) and from whole blood cells using GeneJET Whole Blood RNA Purification Mini Kit (K0761: Thermo Scientific) and reverse transcribed into cDNA (QuantiTect RT kit, Qiagen) according to manufacturer´s protocol and quantitative RT-PCR analyses were performed. Total LMNA expression was determined utilizing primers spanning exon 9 to 12. To specifically quantify progerin expression, primers were designed spanning the splice junction site between exon 11 and 12. Rpl32 was used as reference gene. Exon spanning primers were verified on agarose gels. Sequences of amplification primers were: Rpl32-F: 5´-AGTTCCTGGTCCACAACGTC-3´, Rpl32-R: 5´-CTCTTTCCACGATGGCTTTG-3’, Progerin F: 5’-TCAGGAGCCCAGAGCCCCCAGAAC-3’, Progerin R: 5’-GGGTTATTTTTCTTTGGCTTCA-3’, LMNA-Exon9 F: 5’-GGTGGTGACGATCTGGGCT-3’, LMNA-Exon12 R: 5’-CCAGTGGAGTTGATGAGAGC-3’. The Cycling conditions for qPCR were 95°C for 10 min (Activation), 95°C for 15sec (Denaturation) and 60°C for 1min (Annealing and extension) up to 40 cycles. Using 2x SYBR green mastermix (Applied Biosystems, USA) quantitative gene expression was calculated using the comparative ΔΔCt-method with RPL32 as a reference gene.

### Immunohistochemistry and immunofluorescence

Freshly isolated tissues from human dilated cardiomyopathy, and non-failing control hearts were immediately fixed in 4% neutral buffered formalin. Tissues were embedded in paraffin, and sectioned. Sections of 2 μm thickness were cut and mounted on positively charged glass slides. For Immunohistochemistry staining, deparaffinized sections were incubated with progerin antibody (ab66587, Abcam) followed by Vectastain Elite ABC kit (AK-5000, Vector laboratories, Cambridgeshire, UK) and Vector® Red subtrate kit (SK-5100, Vector laboratories, Cambridgeshire, UK) staining. For Immunofluorescence staining, deparaffinized sections were incubated with primary mouse monoclonal antibodies against progerin (ab66587, Abcam) or (sc-81611, Santa Cruz) overnight at 4°C followed by a one-hour incubation with secondary antibody at room temperature (Alexa Fluor® 488, Molecular Probes Inc, Eugene, OR) and wheat germ agglutinin (WGA) Texas Red™-X Conjugate (W21405, Invitrogen). Sections were co-stained with DAPI to detect all cell nuclei. Digital photographs were taken, and ten random high-power fields (HPFs, 400x) from each heart biopsy sample (n = 3) were analysed utilizing NIH ImageJ software. For quantification, progerin+ nuclei (green) were calculated as percentage to total nuclei DAPI (blue).

Apoptotic cells were detected using the TUNEL assay (DeadEnd™ Fluorometric TUNEL System, Promega) or utilizing an antibody against cleaved caspase-3 (#9661, Cell Signaling). Sections were co-stained with DAPI to detect all cell nuclei. After embedding the section into Vectashield mounting medium with DAPI (Vector Laboratories, Burlingame, CA), the sections were analyzed by Zeiss florescence microscopy and images were acquired with a Zeiss AxioCam (Carl Zeiss Microscopy GmbH, Jena). Digital photographs were taken, and ten random high-power fields (HPFs, 400x) from each heart biopsie sample (n = 3) were analysed utilizing NIH ImageJ software. For quantification, the apoptotic index (AI) was calculated as percentage of TUNEL+ nuclei (green) to total nuclei DAPI (blue).

### Statistical analysis

Statistical analysis was performed using SSPS™ software (V17.0, SPSS Inc., Chicago, USA) and MedCalc (V12.5, MedCalc Software, Belgium). Quantitative variables are expressed as means ± standard deviation (SD), whereas categorical variables are presented as absolute values and percentages. Chi-square tests or an independent T-Test was used to test for differences between the groups. A 2-tailed p-value of less than 0.05 was considered as significant. Correlation coefficients were determined by linear regression analysis (Pearson’s equation) and scatter plots were created. GraphPad Prism (Version 6.04, GraphPad Software, Inc, CA, USA) was used for generation of graphics.

## Results

### Characteristics of hearts

Table 1 shows the characteristics of DCM and control hearts. DCM hearts and control hearts did not significantly differ in age and distribution of sex. There was, however, a significant difference in parameters describing typical measures of heart failure and LV remodeling like ejection fraction (EF), left ventricular endsystolic diameter (LVESD), and left ventricular enddiastolic diameter (LVEDD) between DCM and control hearts. Furthermore, quantitative RT-PCR analyses revealed that mRNA levels of the heart failure marker brain natriuretic peptide (BNP) were significantly upregulated in DCM hearts as compared to control hearts (50.7 ± 10 vs. 4.1 ± 1.6, p≤0.001).

**Table 1:** Characteristics of hearts.

Characteristics	DCM (N = 15)	Control (N = 10)	P Value
Age, years	39.0 ± 9.5	32.5 ± 14.0	0.174
Female sex–no. (%)	3 /15 (20)	4 / 10 (40)	0.295
Ejection Fraction, %	28.5 ± 12.6	58.1 ± 4.0	<0.001***
LVESD, mm	54.2 ± 12.9	31.5 ± 7.1	<0.001***
LVEDD, mm	66.2 ± 9.8	49.9 ± 5.1	<0.001***

Values are mean ± SD.

^*^ P ≤ 0.05

^**^ P ≤ 0.01

^***^ P ≤ 0.001 DCM vs. Control. LVESD: Left ventricular endsystolic diameter, LVEDD: Left ventricular enddiastolic diameter. DCM: Dilated cardiomyopathy, Control: Non-Failing Hearts.

### Progerin mRNA is detectable in human DCM and non-failing hearts

**Fig 1:**
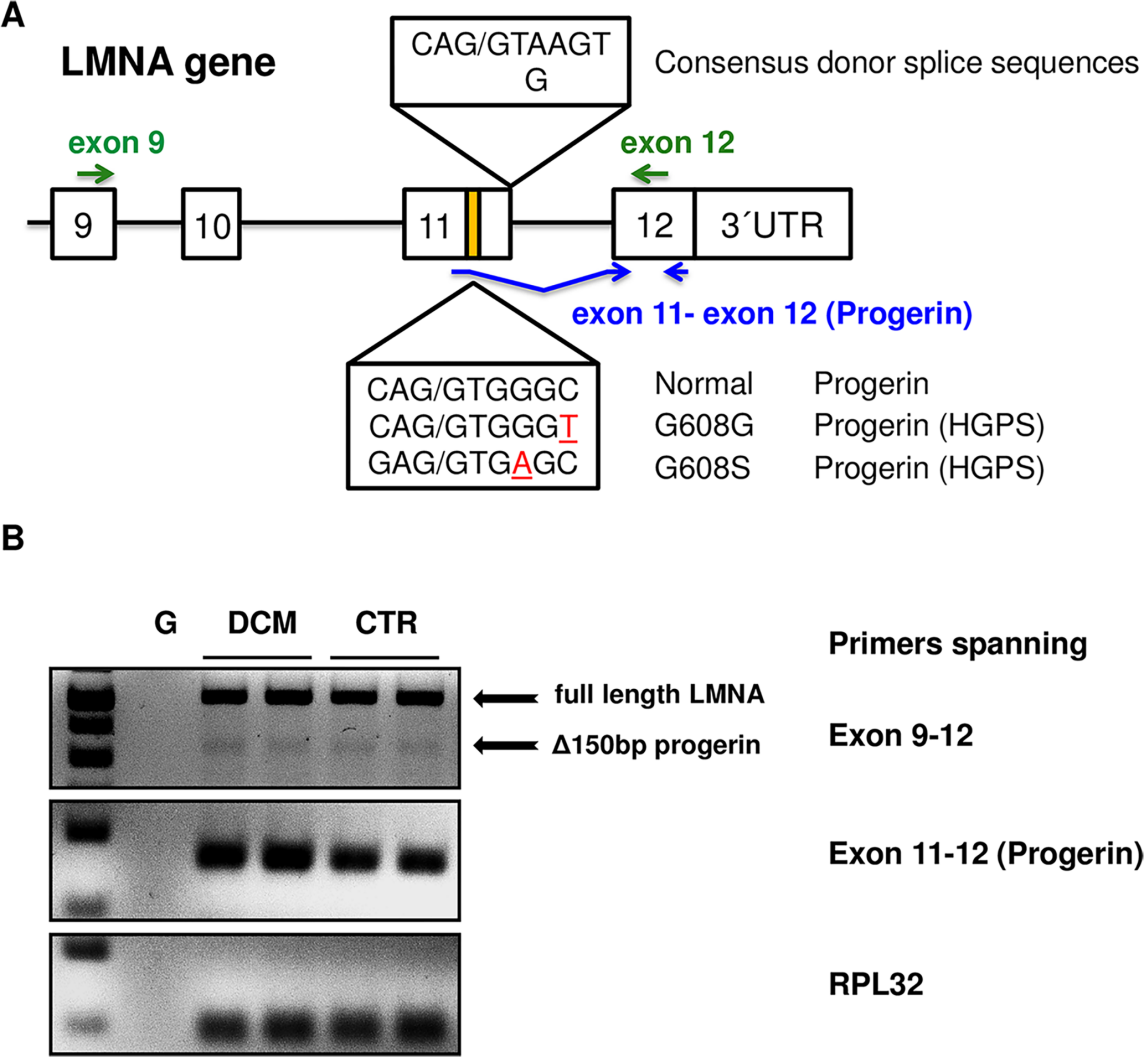
PCR strategy for the detection of progerin expression in heart and blood. **A** Schematic overview of PCR based strategy to analyze progerin mRNA expression in human heart and the blood samples. Shown are the consensus donor splice sequence at the end of exon 11, the sequence of the normal LMNA cryptic splice site, and two common known mutated cryptic splice sites in HGPS patients. **B** First row: PCR gel showing total lamin A (first arrow) and small amounts of progerin expression (arrow showing Δ150bp band). Second row: Specific progerin expression utilizing primers spanning exon 11 to 12. Third row: RPL32 expression was used for relative quantification of progerin expression. G: Genomic DNA (G) did not reveal any significant LMNA bands in the gel verifying specific mRNA expression.

**Fig 2:**
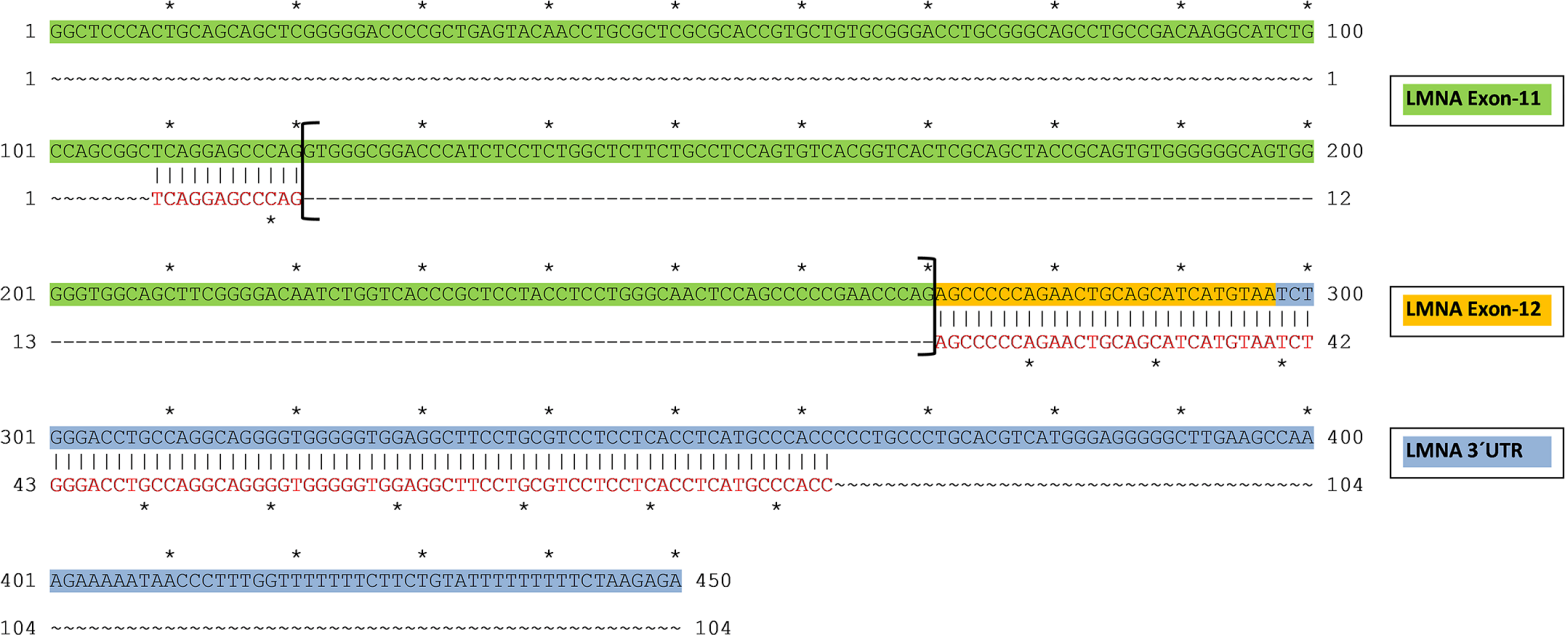
Alignment of progerin PCR product cDNA confirms alternative splicing in the heart. The sequence of purified progerin PCR cDNA (red letters) was aligned to the genomic sequence of LMNA exon 11 (green) to exon 12 (yellow) with the 3´UTR sequence (blue) showing the expected gap of 150bp between exon 11 to exon 12 confirming alternative splicing of progerin.

Fig 1A depicts the schematic PCR based strategy to analyze progerin mRNA expression in human heart and the blood samples. Shown are the consensus donor splice sequence at the end of exon 11, the normal LMNA progerin cryptic splice site, and two common (> 90%) mutated cryptic splice sites in HGPS patients. LMNA expression was determined utilizing primers spanning exon 9 to 12 to detect both the full-length LMNA and the truncated progerin isoform and primers specifically designed to detect the amount of truncated progerin expression. Fig 1B (first row) shows representative gel bands derived from c-DNA samples of DCM hearts and non-failing controls amplified with primers spanning exon 9 to 12 depicting both the full-length LMNA (upper arrow) and the truncated progerin isoform (arrow showing Δ150bp band, upper row). Fig 1B (second row) reveals that the progerin splice variant was readily detectable in human heart samples derived from DCM patients and non-failing hearts. Samples with genomic DNA (G) did not reveal any significant LMNA bands in the gel verifying specific mRNA expression (last row). Further sequence alignment of the purified specific progerin PCR product from DCM hearts revealed the anticipated gap of 150bp between exon 11 and 12 of the LMNA gene sequence confirming alternative splicing of progerin in the heart (Fig 2).

### Progerin mRNA is upregulated in DCM hearts

**Fig 3:**
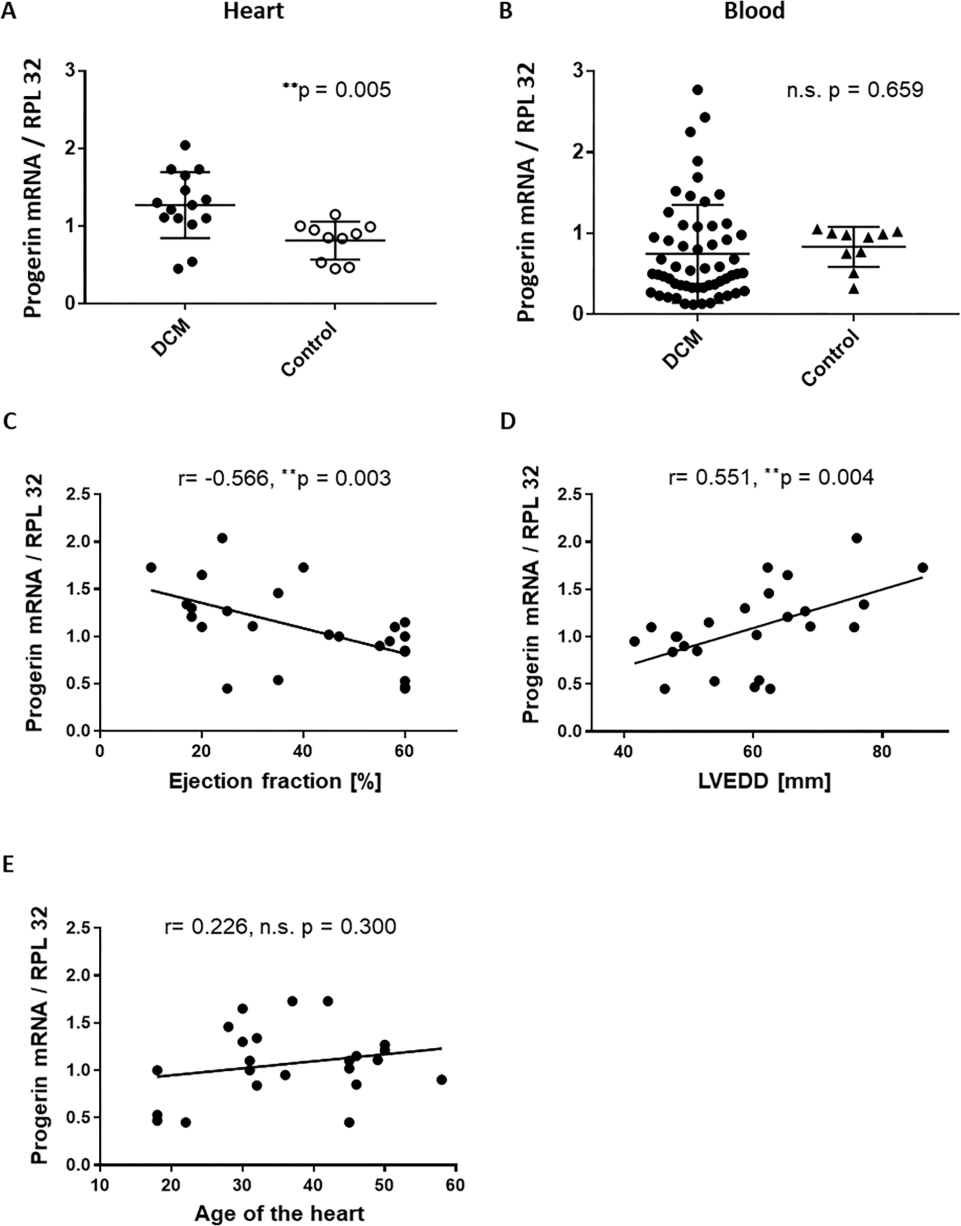
Progerin mRNA is upregulated in DCM hearts and is significantly correlated with measures of heart failure but not with age. **A** Relative amount of progerin mRNA levels related to the reference gene RPL32 in heart biopsies of patients with DCM (n = 15) and non-failing control hearts (n = 10). Shown are all individual data points, lines show mean ± SD. ^**^ P ≤ 0.01 DCM vs. Control. **B** Relative amount of progerin mRNA levels related to the reference gene RPL32 in whole blood cells derived from patients with DCM (n = 56) and healthy controls (n = 10). Shown are all individual data points, lines show mean ± SD. P-value was derived from unpaired two-sided T-Test between the groups. **C** Scatter plot showing the negative correlation between the relative amount of progerin mRNA related to RPL32 with ejection fraction in human heart biopsies (n = 25). ^**^ P ≤ 0.01. **D** Scatter plot showing the positive correlation between the relative amount of progerin mRNA related to RPL32 with left ventricular enddiastolic diameter (LVEDD) and **E** Scatter plot showing the positive correlation between progerin mRNA related to RPL32 with the age of the hearts in human heart biopsies (n = 25). N.s. not significant. ^**^ P ≤ 0.01.

As shown in Fig 3A, RT-PCR analyses of samples from DCM hearts (n = 15) revealed a significant upregulation of progerin mRNA as compared to non-failing controls (n = 10) (1.27 ± 0.42 vs. 0.81 ± 0.24; p = 0.005). In contrast to the heart, progerin mRNA isolated from whole mononuclear blood cells of DCM patients (n = 56) or healthy controls (n = 10) did not reveal significantly different progerin levels as revealed by unpaired T-Test analysis (0.75 ± 0.60 vs. 0.83 ± 0.24; p = 0.661 between the groups; Fig 3B). Taking our data of progerin expression in the heart into account a statistical power calculation predicts a power of 93.5% for the detection of differences in the expression of progerin between DCM hearts and controls (α≤0.05, two-sided, for details see also https://www.dssresearch.com/KnowledgeCenter/toolkitcalculators/statisticalpowercalculators.aspx. Since our results are highly significant our data could provide the rational for sample size calculations in further clinical studies investigating the effect of progerin in the heart failure population.

### Progerin highly correlated with measures of LV remodeling

We further performed linear regression analyses based on Pearson’s correlation to calculate whether progerin mRNA levels were correlated to measures of LV remodeling. As shown in Fig 3C and 3D linear regression analysis revealed that progerin mRNA is significantly negatively correlated to EF (r = -0,567, p = 0,003) and positively correlated to LVEDD (r = 0,551, p = 0,004). As shown in Fig 3E progerin mRNA levels were positively related to the age of heart, however this correlation was not significant (r = 0.227, p = 0.300) suggesting that rather “myocardial aging” was correlated with progerin mRNA levels.

### Progerin levels were not influenced by inflammation in DCM hearts

Since we included 6 DCM hearts with a histologically confirmed diagnosis of inflammatory cardiomyopathy we compared progerin mRNA levels in idiopathic and inflammatory DCM hearts. As shown in Table 2 there was no significantly difference between inflammatory status and progerin levels in the heart. Further analyses did also not reveal any significant correlation of progerin mRNA levels with CD45+, CD3+, and CD14+ cells in the heart suggesting that other mechanisms influence progerin expression.

**Table 2:** Progerin and inflammation in the heart.

parameter	No inflammation (N = 9)	Inflammation (N = 6)	P Value
progerin mRNA	1.36 ± 0.43	1.12 ± 0.41	0.315
CD45+ cells/mm2	7.6 ± 1.7	26.0 ± 9.9	0.006**
CD3+ cells/mm2	4.9 ± 1.9	15.0 ± 7.8	0.004**
CD11c+ cells/mm2	7.1 ± 2.2	12.3 ± 4.0	0.019*

Values are mean ± SD.

^*^ P ≤ 0.05

^**^ P ≤ 0.01 no inflammatory CMP vs. inflammatory CMP.

### Increased numbers of progerin+ and TUNEL+ cells in DCM hearts

**Fig 4:**
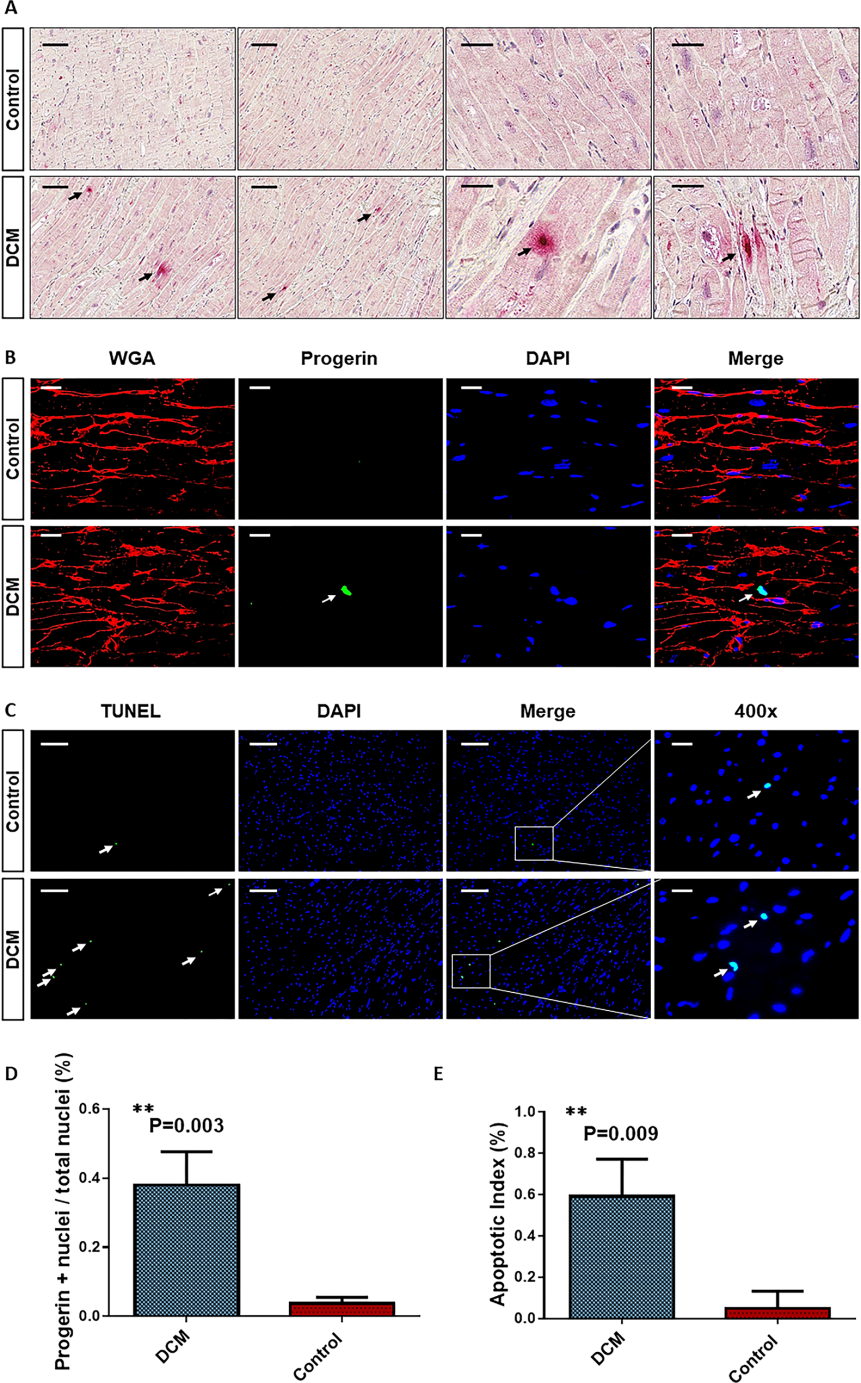
Increased progerin expression and TUNEL+ cells in DCM hearts. **A** Immunohistochemistry showing increased numbers of progerin expressing cells (arrows, red nuclei) in DCM hearts compared to non-failing controls. Scale bar represents 100μM (rows 1–2) and 25μM (rows 3–4). **B** Immunofluorescence stainings revealed increased expression of progerin (green, arrows) in nuclei (blue) of DCM hearts (merged images last row, arrows) compared to non-failing controls. Red: WGA membrane staining, Green: Progerin staining, Blue: DAPI+ nuclei. Scale bar represents 20μM. **C** TUNEL staining of apoptotic cell death revealed increased numbers of TUNEL+ cells in DCM hearts compared to non-failing controls. Scale bar represents 200μM (rows 1–3) and 20μM (last row). **D** Quantification of progerin+ nuclei to total nuclei per high power field (HPF) in DCM hearts and Controls (n = 3). ** P ≤ 0.01 DCM vs. Control. **E** Quantification of apoptotic index (TUNEL+ nuclei to total nuclei) in DCM hearts compared to controls (n = 3). ** P ≤ 0.01 DCM vs. Control.

**Fig 5:**
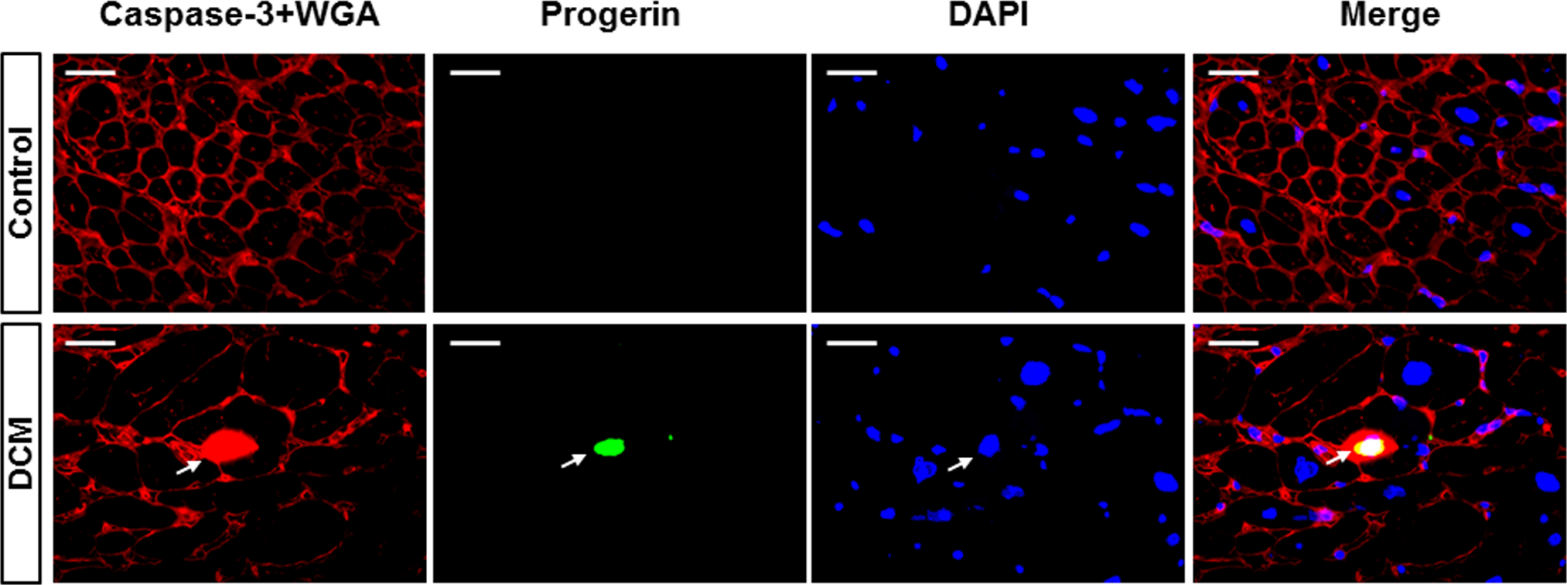
Co-expression of apoptotic marker caspase-3 and progerin in DCM. Immunofluorescence stainings revealed expression of the apoptotic cell death marker cleaved caspase-3 (red, arrow) and progerin (green, arrow) in the nucleus (blue) of a cardiomyocyte delineated by red cell membrane staining with WGA (merged image last row, arrow) compared to non-failing controls. Scale bar represents 20μM.

Next, we determined progerin expression by immunohistochemistry and immunofluorescence in DCM as well as in human non-failing hearts. As shown in Fig 4A, immunohistochemistry revealed progerin expression in the nucleus of most likely cardiomyocytes, while non-failing hearts showed almost no progerin+ cells. Immunofluorescence staining against the cell membrane with WGA (red), specific antibodies against progerin (green, arrows) and nuclei (DAPI, blue) also revealed an increased expression of progerin protein in cell nuclei of DCM hearts (bright blue, merged image, arrows, Fig 4B), whereas healthy donor hearts did not reveal sustained progerin expression (Fig 4B, first line). Further quantification confirmed significantly increased numbers of progerin+ nuclei to total nuclei in DCM hearts compared to non-failing hearts (Fig 4D). Additional TUNEL staining for apoptotic cell death in heart sections derived from DCM hearts with increased progerin+ cells revealed a significantly increased apoptotic index (TUNEL+ nuclei to total DAPI+ nuclei) of TUNEL+ cells as compared to controls (Fig 4C and 4E). In order to elucidate whether progerin+ cells are directly linked to apoptotic cell death, we performed co-stainings for cell membrane (WGA, red), the apoptotic cell death marker cleaved caspase-3 (red nucleus) progerin (green) and nuclei (DAPI, blue). In contrast to control hearts, were able to detect caspase-3+/progerin+/DAPI+ nuclei (merged image) in cardiomyocytes (Fig 5).

## Discussion

Here we provide first experimental evidence that progerin, associated with premature aging in HGPS is upregulated in human DCM. Progerin mRNA expression in the heart was strongly significantly correlated with left ventricular remodeling. Although there was a weak positive correlation between age and progerin mRNA expression, statistical testing revealed no significant differences. These data suggest that not the age of the heart per se but rather the process of “myocardial aging” defined by a progressive deterioration in cellular and organ function with time [18] is associated with increased levels of progerin mRNA. The narrow age range of hearts from 18 to 58 years in our study could also be a reason that statistical significance was not reached. Progerin highly attracted attention because it accumulates also in non-mutated ageing human mesenchymal stem-cells [4], in dermal fibroblasts, terminally differentiated keratinocytes [22]. It is known that prelamin A accumulation plays a key role in aging in several tissues, including the vasculature and is discussed as a marker for vascular aging [25]. However, it is currently unknown whether this is relevant in myocardial aging, too [26]. Since LMNA gene mutations are causally involved in patients with idiopathic dilated cardiomyopathy (3.6%) and familial dilated cardiomyopathy (7.5%) [27], we hypothesized that accumulation of progerin in non HGPS´s individuals in the heart may as well be involved in the progression of DCM. To our knowledge we show for the first time that progerin is upregulated in human DCM hearts suggesting that accumulation of progerin (prelamin A) could be involved in the progression of DCM and myocardial aging. Aging is defined as a biological process leading to deterioration of cellular and organ function with time [18]. In line with this definition increased progerin mRNA levels in the heart were significantly correlated to reduced heart function (EF) and LV remodeling (LVEDD) assuming that progerin is involved in structural changes in the heart and deterioration of function.

Previous studies revealed that expression of progerin is associated with severe nuclear defects like disturbed lamina, sustained DNA and telomere damages, leading to early cellular senescence, apoptosis and finally to impairment of organ function [18, 28] supporting our findings that increased levels of progerin+ nuclei were associated with increased numbers of apoptotic TUNEL+ cells in DCM hearts. Moreover, we were able to detect co-staining of the apoptosis marker cleaved caspase-3 and progerin in nuclei of cardiomyocytes in DCM hearts suggesting a direct link of progerin to apoptotic cell death in the heart. Thus, negative effects of progerin expression could have led to the loss of contractile heart tissue accompanied by disease progression and enlargement of the ventricle. Our findings are further supported by murine models showing that accumulation of prelamin A led to the development of DCM [29]. In contrast to the heart, we found no significantly differences of progerin mRNA expression in isolated blood cells. These findings might be explained by the fact that mononuclear blood cells have a limited life span of several days so that cumulative defects of alternative splicing might not penetrate or because lamin A/C is not found in some differentiated hematopoietic blood cells [30]. In contrast, the heart is known to be a solid organ with a very low turnover rate of cardiomyocytes [31]. Thus, progerin might accumulate over time and once a critical amount is reached, heart cells could suffer damage and i.e. undergo senescence or apoptosis. A recently published study supports the assumption that alternative splicing increases with aging and could lead to progression of disease [32]. Another finding in our study was that inflammatory cardiomyopathy was not linked to increased progerin levels suggesting that inflammation was not the trigger for the increased expression of progerin seen in DCM hearts.

Finally, the establishment of new therapeutic approaches for heart failure treatment could be an achievable goal since mTOR inhibitors like rapamycin have been described to increase cardiac function and survival in lamin A/C-deficient DCM mice by inhibiting mTORC1 signaling and lowering progerin levels in cell culture [33, 34]. Another therapeutic concept could be the strategy to increase lamin C at the expense of the progerin precursor prelamin A since both products are derived from alternative splicing of LMNA. Regarding the latter, antisense oligonucleotide mediated knockdown of the serine/arginine-rich splicing factor 2 regulating alternative splicing of LMNA decreased progerin expression in HGPS mice [35]. Taking our new findings in human DCM into account these suggest a potential treatment strategy for progerin related DCM and heart failure.

### Limitations of the study

Since we have not performed genetic sequencing of the lamin A gene in patients suffering from DCM we cannot rule out the possibility that lamin A mutations may have influenced the expression of progerin. However, since lamin A mutation only account for ca. 6% of DCM cases, this could not explain the commonly seen upregulation of progerin in DCM hearts compared to non-failing controls. Moreover, we cannot entirely exclude the possibility that progerin is secondarily upregulated in DCM rather than being involved in etiology and progression of the disease. Therefore, animal models will be necessary to further clarify the mechanisms regarding the role of progerin in DCM pathology.

## Conclusions

Our data indicate that the aging related splice variant progerin is upregulated in human DCM hearts and correlates with LV remodeling suggesting a role in the progression of heart failure. This hypothesis should be further evaluated in suitable animal models.
